# Barriers and Facilitators of Breast Cancer Screening amongst Culturally and Linguistically Diverse Women in South Western Sydney: A Qualitative Explorative Study

**DOI:** 10.3390/ijerph18179129

**Published:** 2021-08-30

**Authors:** Javeria Jamal, Freya MacMillan, Kate A. McBride

**Affiliations:** 1 School of Medicine, Western Sydney University, Sydney, NSW 2560, Australia; 18067343@student.westernsydney.edu.au; 2 Translational Health Research Institute, Western Sydney University, Sydney, NSW 2560, Australia; F.Macmillan@westernsydney.edu.au; 3 School of Health Science, Western Sydney University, Sydney, NSW 2560, Australia

**Keywords:** breast screening, mammogram, CALD women, health promotion

## Abstract

Breast cancer is the most common cause of cancer amongst Australian women and the second most common cause of cancer mortality. Despite the proven effectiveness of early intervention, screening rates remain subpar across many regions in New South Wales (NSW). Screening rates are particularly low within the culturally and linguistically diverse (CALD) area of South Western Sydney (SWS). The objective of this study was to qualitatively explore barriers and facilitators to breast screening from the perspectives of CALD women from SWS. CALD women aged ≥40 who resided in SWS were invited to participate in a semi-structured interview to explore barriers and facilitators to breast cancer screening. Interviews were recorded, transcribed verbatim and analysed thematically to identify recurring patterns in the data. Sixteen women from CALD backgrounds participated. Women in this study reported absence of symptoms, fatalistic beliefs and embarrassment during the procedure to be the primary reasons for reluctance to screen. Lack of general practitioner (GP) endorsement, transport issues and pain associated with the procedure were also reported as additional barriers to screening. Common facilitators to screening included encouragement from family and friends, family history of cancer and media adverts. CALD women have distinctive barriers to mammography, which lead to poor breast screening participation rates. Opportunistic health promotion in this area is warranted and may lead to better health outcomes amongst this population.

## 1. Introduction

Breast cancer is the most common cancer among Australian women, representing 28% of all cancers, and is the second highest cause of cancer related mortality amongst females [1,2,3]. By age 85, Australian women have a ~1 in 7 risk of diagnosis [1]. Treatment is costly, with over AUD 800 million spent on therapy in 2013 [4].

An effective way to mitigate some of the health and financial impacts of breast cancer is through screening programs [4,5]. BreastScreen Australia offers free biennial mammograms to women aged 50–74 and a sharp decline in breast cancer-related deaths has been recorded since the introduction of this program [5,6]. Several studies have demonstrated that mammograms are effective in recognising early-stage cancer, while others found a proportion of late-stage cancers are attributable to failure to participate in screening [7,8,9]. If breast cancer is detected through screening programs, Australia’s universal healthcare scheme “Medicare” covers the cost of treatment, thereby ensuring access and affordability of care [10,11]. Despite the effectiveness and affordability of early detection and intervention, mammographic participation rates are suboptimal, with ~60% of women aged 60–69 and only 50% of women aged 50–54 screening in 2015–2016, well below BreastScreen Australia’s target rate of 70% [2,11,12]. South Western Sydney (SWS) has a very low screening rate of 46.3%, ranking it the lowest in NSW [2,13]. Therefore, a number of eligible women with potential malignancy may be diagnosed when their disease has progressed, with resultant poorer outcomes [14].

One reason for this particularly low participation rate may be the region’s large culturally and linguistically diverse (CALD) population, with ~52% of the population born outside Australia and 64% speaking a language other than English at home [15,16,17]. No studies have specifically examined this region, with only a few attempting to explain these across the broader Australian population [18,19,20,21]. These studies reported that CALD women were less aware of cancer screening and more likely to report not understanding the procedure purpose, with only 62.9% of Arabic women reporting they had heard of mammography [19,21]. Women also reported they were “feeling well” or had “no family history”, meaning there was no need to screen, reinforcing the importance of community education about the need for screening [13,19].

Even when CALD women do understand the mammographic procedure, they may avoid it because of cultural beliefs and values, including modesty and fatalistic beliefs, or due to limited English proficiency [13,18,20]. CALD women also report similar issues to the general population, with time constraints and appointment availability both reported barriers to screening [13,21].

No studies examining barriers and facilitators to screening participation have been conducted among the multicultural population of SWS, where screening remains low despite learnings from research elsewhere in Australia with CALD women [19,20,21]. Investigation with women from this highly multicultural area is warranted to investigate reasons for low screening rates. The objective of this study was to qualitatively explore barriers and facilitators to breast screening from the perspectives of CALD women from SWS, and ultimately, develop solutions for the public health issue of suboptimal rates of screening attendance in CALD populations.

## 2. Materials and Methods

### 2.1. Study Design

A qualitative approach was selected to allow participants to discuss in-depth the barriers and facilitators that influence participation in timely breast screening [22]. This approach allowed researchers to participate in naturalistic conversation, understand perspectives and generate descriptive narratives [23].

### 2.2. Participants and Recruitment

Inclusion criteria were women ≥40 years of age eligible for the BreastScreen program, who were from a CALD background (defined as those who were born overseas and spoke a language other than English at home) and resided in SWS [24].

Recruitment occurred through distribution of recruitment flyers at community health facilities and at community events. Snowball sampling was used, where contacts of initial participants were leveraged to recruit new additional participants meeting the eligibility criteria.

### 2.3. Data Collection

Participants were contacted by a member of the research team (JJ) to arrange an in-depth, one-on-one, 30–45 min semi-structured interview either in person or via telephone. Written, informed consent was sought before the interview. A semi-structured interview schedule was developed from a previous study that investigated perspectives on breast screening amongst women who self-identified as obese [25]. The interview schedule included questions on previous breast screening participation, including barriers to screening, screening experiences and knowledge surrounding the purpose of mammography (Appendix A).

### 2.4. Analysis

Interviews were digitally voice recorded, then transcribed verbatim. Transcripts were de-identified with any identifiable information (e.g., workplace, address) being replaced with pseudonyms. Data were coded in Quirkos (https://www.quirkos.com accessed on 19 February 2019 (Quirkos, Edinburgh, UK), a software tool designed for management of qualitative text by JJ. Coded data were then clustered and grouped into broader themes and categories, which were regularly revisited by the whole research team (JJ, FM and KM). Participant excerpts were arranged in tables according to themes/categories and labelled by the women’s CALD background, age and participant number (e.g., Lebanese, 53, P1 = A Lebanese woman aged 53 years who was participant number 1).

### 2.5. Ethics Approval

Ethical approval for this study was provided by the Western Sydney Human Research Ethics Committee (H12805). Written consent was obtained from participants before commencing interviews.

## 3. Results

### 3.1. Participants

Sixteen women participated, including 11 women who had previously attended breast screening. Participant demographics are outlined in Table 1.

Recurring themes were classified into several categories: (1) healthcare and illness-related experiences influencing screening; (2) cultural barriers towards screening attendance; (3) personal barriers towards mammography; and (4) general facilitators to screening. These categories are now explored.

**Figure 1:**
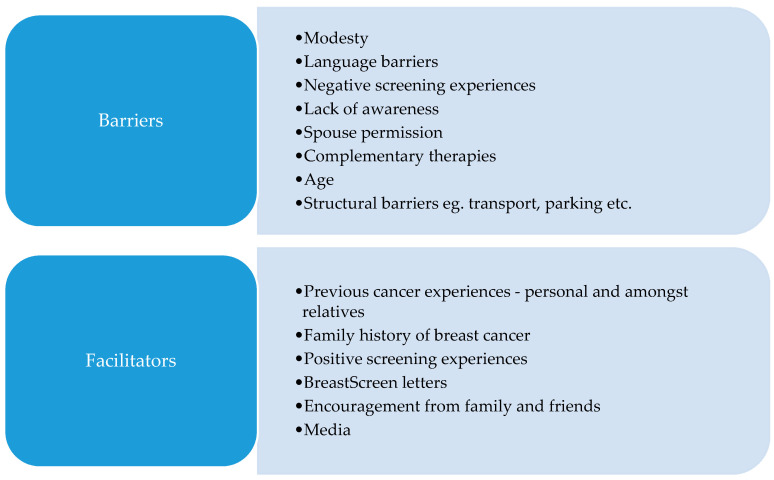
Summary of barriers and facilitators.

A summary of barriers and facilitators is provided in Figure 1.

### 3.2. Healthcare and Illness-Related Experiences Influencing Screening

Almost all (88%) participants cited that previous healthcare experiences played an important part in their decision to screen (as per Table 2). Personal cancer experiences or those of family and friends were identified by participants as motivators to screen. Several women related how watching their friends experience breast cancer reinforced the importance of screening to detect cancer as early as possible (excerpts 2.1 and 2.2). One participant, however, outlined the experience of having a neighbour who was diagnosed with late-stage breast cancer despite having regular mammograms as being a deterrent towards mammography (excerpts 2.3). The participant reported that this experience had overwhelmed her with feelings of helplessness in the face of cancer so that she no longer felt motivated to attend screening. Many women identified that having a family history of cancer also encouraged them to attend screening as they were aware that they were at increased risk of breast cancer (excerpts 2.4 and 2.5). One participant, however, mentioned that a lack of family history was the reason she consciously chose to delay screening (excerpt 2.6).

Multiple women described positive experiences of the procedure itself, with these primarily attributable to the professionalism and sensitivity of staff conducting mammograms (excerpt 2.7). Two women, however, reported the “rough” nature of particular staff members as being a barrier to reattending future screening (excerpts 2.8 and 2.9). Several women also described mammograms as being physically uncomfortable; however, most agreed the discomfort was tolerable and would not prevent them from screening again.

### 3.3. Cultural Barriers towards Screening Attendance

Most (75%) participants highlighted the importance of cultural factors in influencing screening practices (as per Table 3). Several women emphasised the importance of maintaining modesty in their culture and expressed embarrassment over undressing for a mammogram (excerpts 3.1, 3.2 and 3.3). These women acknowledged that this discomfort and embarrassment caused them to delay screening appointments (excerpt 3.4). An overall preference for female radiographers over male radiographers was reported by multiple women, with many stating that the all-female staff at BreastScreen made them feel more comfortable during mammograms (excerpt 3.5).

Most women in this study agreed language barriers were not an issue towards screening. Participants said information was available in their language and if needed, children accompanied them for interpretation (excerpts 3.6 and 3.7). One participant, however, expressed that her minimal English skills prevented her from completely understanding the procedure (excerpt 3.8).

Low health literacy was also apparent among several participants and appeared to influence their attendance at screening programs. For example, one participant mentioned that whilst she is reasonably proficient in English, she found the medical jargon associated with screening difficult to comprehend (excerpt 3.9). Another woman said her low knowledge surrounding mammograms was likely due to mammograms not being performed in her home country. Further, several women said that as they currently have no symptoms, they were not required to attend mammograms (excerpt 3.10).

Many women said there was a general lack of awareness of screening within their community and that there was a need to directly deliver the message to these women either through cultural events or via general practitioners (GPs) (excerpts 3.11 and 3.12).

The issue of having to gain permission from her husband to screen was also raised by one participant. The participant mentioned that a discussion with her husband and his approval were mandatory before she could participate in mammography (excerpt 3.13).

Distrust in Western medicine was raised as a cultural barrier towards screening, with several women preferring the use of complementary therapies and natural medicine to prevent breast cancer (excerpt 3.14).

### 3.4. Personal Barriers towards Mammography

Several personal barriers were outlined by women as reasons for why they found screening challenging (as per Table 4). One woman mentioned that a blood clotting disorder diagnosis prevented her from screening (excerpt 4.1) with the fear of bleeding post-mammogram leading her to be cautious of screening as it may result in illness or hospitalisation. Another participant reported that normal results in previous mammograms had caused her to stop attending future mammograms (excerpt 4.2). Many of these women reported performing self-exams, stating that they would only consider screening if they found something suspicious (excerpts 4.3 and 4.4). Only one participant recognised that a self-exam could not wholly substitute examination by a medical professional or formal imaging (excerpt 4.5).

Several women also cited old age as a reason to stop screening (excerpt 4.6). Such participants embraced the idea that screening was useful when they were young, but no longer required it as death was inevitable. Some women reported that fear of being diagnosed with breast cancer and being confronted with thoughts of death had caused them to delay screening (excerpts 4.7 and 4.8). Many women in this study outlined fatalistic beliefs regarding the inevitability of death to be the reason why they considered screening unnecessary (excerpt 4.9).

Multiple structural barriers were also identified by women to be the reason why they found it difficult to screen. Some said it was difficult to find parking around the BreastScreen clinic, while others reported they could not drive and did not want to bother others to take them (excerpts 4.10, 4.11 and 4.12). Two women reported that many of their family and friends found it difficult to make time for screening in their busy daily schedules (excerpts 4.13 and 4.14).

### 3.5. General Facilitators to Screening

Participants outlined a number of factors that encouraged them to screen for breast cancer (as per Table 5). For example, all of the study participants reported the importance of their general practitioner in firstly convincing them to screen and reminding them when mammograms were due (excerpts 5.1, 5.2 and 5.3). Many women said the letters sent from BreastScreen reminded them to screen; however, most agreed they would have eventually screened regardless (excerpts 5.4 and 5.5). For women who had already chosen not to screen, these reminders neither encouraged nor changed their opinion on screening.

Support from family and friends also facilitated screening. Many women reported that having discussions with friends about breast cancer and screening helped to remind them to attend mammograms regularly. Several women reported that reminders from their children or siblings motivated them to screen (excerpts 5.6, 5.7 and 5.8). One woman said the principles of looking after your health were enforced by her parents at a young age, which motivated her to have mammograms (excerpt 5.9). Social support from peers was also mentioned as a facilitator. Some women outlined that screening as a group with friends would alleviate some of their anxiety around screening and encourage them to go regularly (excerpt 5.10).

Several women said that media around screening such as adverts from BreastScreen encouraged them to participate. Many women mentioned that hearing stories of young celebrities, such as Kylie Minogue, being diagnosed with cancer, encouraged them to screen (excerpt 5.11). Some participants felt media could play a bigger role in encouraging breast screening by sharing the stories of regular people diagnosed with cancer (excerpt 5.12). These participants said by having regular people share stories, the experience would be more relatable and have a stronger influence on mammogram attendance. Some women also suggested that social media could be useful in raising awareness of screening among younger women, who could then persuade their mothers to screen (excerpts 5.13, 5.14 and 5.15).

## 4. Discussion

Our study aimed to qualitatively investigate the barriers and facilitators to breast screening among culturally and linguistically diverse women in the highly multicultural region of SWS. Numerous barriers and facilitators that influence participation in breast screening within this community were identified.

Several facilitators were common to women in the general population. These included encouragement by friends and family, the media and having a regular doctor [26]. Awareness of increased cancer risk due to a previous history of breast problems or positive family history of breast cancer found here were also cited as facilitators to breast screening previously [26]. Common barriers identified included lack of time to attend appointments, fear of the pain of the procedure and lack of awareness of the service and how to access it [27].

As well as these common facilitators and barriers, several distinctive issues were also raised. Most women we spoke to expressed feelings of embarrassment around exposing their breasts for a mammogram and cited the importance of modesty within their culture as a factor for reluctance to screen. While some of these women identified having all-female staff at BreastScreen was sufficient to make them feel comfortable, others indicated that the procedure was humiliating regardless of the radiographer’s gender. Embarrassment and discomfort often led to negative screening experiences, which were occasionally compounded due to “rough” and insensitive physical manhandling by staff. These findings are similar to existing literature examining mammography utilisation by minority women in the United States (US) and research conducted amongst different cultural populations in South America [28,29,30]. It is therefore important to recognise the embarrassment experienced by many CALD women during mammograms, even when conducted by a female radiographer. A strategy to address this discomfort may be to increase discussion of screening in social groups and GP consults to normalise and increase familiarity with the procedure. Cultural competency training among radiographers may also be warranted.

Several women in this study had a limited understanding of mammography, which subsequently influenced their screening participation. Many women perceived they were not required to screen as they were asymptomatic, or that self-examination could replace formal screening. This finding indicates that both healthcare providers and media advertisements need to emphasise the importance of attending screening irrespective of symptoms and self-examination. This lack of understanding of the procedure was also exacerbated for women in our study due to language barriers and the fact mammograms were not performed in their home country. While all our participants could speak and understand basic English, the medical jargon associated with screening was difficult to comprehend, particularly for women that had never experienced mammograms. This association between suboptimal English proficiency and low breast screening rates is consistent with previous research about CALD women’s screening practices [29,31]. Overall, these findings suggest a culturally sensitive approach is required to increase awareness of the importance of mammograms among CALD communities. A possible strategy may be to deliver messages directly to communities through social events and culturally competent health support workers, ensuring that the message is understood. An approach may be to recruit and train women who already participate in screening as community social workers, in order to support and change the attitude of other women towards screening.

Another novel finding of this study was that encouragement by a GP was crucial in supporting CALD women to screen. Women perceived GPs as influential figures vital for informing them and reminding them to screen. Several women indicated advice from their GP was valued above media advertisements and health promotion campaigns, suggesting that promoting screening in these communities requires direct encouragement from GPs rather than generalised campaigns. A simple strategy could be a primary care tool to prompt reminders for mammograms during consults. The essential role of physicians in recommending screening amongst CALD women is supported by previous studies and may be due to the perception of GPs as authoritative figures, essential in assisting communities in navigating healthcare systems [31]. Research exploring general practitioner perspectives on providing mammographic advice to CALD communities is warranted.

An important finding from this study was that fear and fatalistic beliefs amongst CALD women led to avoidance of mammography. Many women expressed fear of being diagnosed with cancer, due to repercussions of a cancer diagnosis on their lives and their family. Several participants perceived cancer as unavoidable, untreatable, and preferred not to engage in screening as it was considered futile. Women of older age groups expressed that screening was no longer necessary due to the inevitability of death. The issue of fear and fatalistic beliefs interfering with timely mammography is well supported in previous studies investigating screening behaviours of CALD women [32,33,34]. A possible strategy to address fear and anxiety around screening involves encouraging CALD women to attend group mammograms. Participants have indeed indicated that going to BreastScreen with friends would allow them to feel more supported and encouraged to continue screening. Increased awareness about the benefits of early detection and treatment may also serve to increase screening rates amongst this population.

Based on our study findings, several targeted changes to health promotion and clinical practice should be considered for CALD women. These include sensitivity training for radiographers, emphasising strategies such as minimising exposure of the breasts, providing privacy to dress/undress and explaining the procedure throughout the screening process. GPs should also be advised to regularly discuss mammograms in consultations and provide clear instructions on how to access BreastScreen vans. Media campaigns on cultural radio stations and TV channels should convey information about the high cure rates associated with early detection and intervention. Culturally competent healthcare workers should also be trained to deliver messages to their respective communities at large social events.

A strength of our study was that it provided participants with an opportunity to openly discuss screening in-depth. Interviews were conducted by a female interviewer of Pakistani background, allowing participants to feel more comfortable in relating their experiences. However, our sample consisted primarily of women who were of eastern European background and reasonably proficient in English, thereby limiting representation of women from differing backgrounds and those who do not speak English well. Further, this was a small qualitative study with results not generalisable. Nonetheless, our sample size was sufficient to reach data saturation and has highlighted several key issues for CALD women around breast screening.

## 5. Conclusions

While many barriers and facilitators to breast screening among CALD women also exist in the general population, CALD women raised several additional issues. Our findings highlight numerous health promotion strategies to encourage breast screening amongst ethnic populations. These include increased GP recommendation through the introduction of primary care tools to prompt reminders for mammograms during consults, cultural competency training among radiographers and direct engagement with CALD communities via staff of similar cultural backgrounds.

## Figures and Tables

**Table 1:** Demographic characteristics of participants.

Women Interviewed	*n* = 16
Age group	
50–54	2 (13%)
55–59	3 (19%)
60–64	1 (6%)
65–69	3 (19%)
70–74	5 (31%)
75–79	2 (13%)
Mean age	65 ± 8.0
CALD background	16 (100%)
Italian	8 (50%)
Lebanese	1 (6%)
Maltese	2 (13%)
Filipino	1 (6%)
Croatian	1 (6%)
Fijian	1 (6%)
Uruguayan	1 (6%)
Pakistani	1 (6%)
Screening history	
Regular Screeners	10 (63%)
Lapsed Screeners	1 (6%)
Never Screeners	5 (31%)

**Table 2:** Participant-reported healthcare and illness-related experiences.

Emerging Theme	Excerpt Number	Excerpt
Cancer experiences	2.1	Sometimes through unfortunate experiences that women have gone through, it makes you stop and think as if—“No, I should still continue.” Because myself, I’ve had quite a few thoughts of like, “Oh, no, I’m not gonna do it anymore. What for? Wasting time, it’s uncomfortable.” We tend to have a lot of negativities in ourselves not to do it, but then when you hear an incident and it’s someone that you’ve dealt with for a long time, it sort of makes you change your way of thinking as if like, “No, I better continue”. (Italian woman, 57—Participant 2)
	2.2	....you hear friends having a problem with… this friend of mine, she’s in her 50s and she’s…gone through a chemo and all that and so you know that it’s an important thing to do. (Italian woman, 70—Participant 8)
	2.3	My neighbour, she’d done it [mammograms] and a couple of weeks later, she died from breast cancer. So what’s the point? They’re missing it already. (Lebanese woman, 53—Participant 1)
	2.4	My father had a bowel cancer, so I thought, “What if I have this sort of gene?” after losing my period so young and I have hysterectomy, [at] 36. (Italian woman, 67—Participant 5)
	2.5	I think lot of women, if they have… a family history of high risk in these malignant things, then of course… You would do it without even hesitation. (Italian woman, 57—Participant 2)
	2.6	I’m not gonna do... I’m not concerned at all because my history of my family… we don’t have cancer in the family at all. (Lebanese woman, 53—Participant 1)
Screening experiences	2.7	I can’t fault the staff there that… do these tests. They’re very professional. …They know that it’s uncomfortable and …they’ve always—try and make you feel as comfortable as possible. (Italian woman, 57—Participant 2)
	2.8	She wasn’t understanding that I was hurting. She said, “Gee, you’re a whinger,” and I thought, “Excuse me.” I said, “It hurts.” She says, “You’re not the only one.” I know I’m not the only one, but it hurts. (Italian woman, 67—Participant 5)
	2.9	No one is doing it in Liverpool because…. they do it rough. …and it doesn’t matter how many letters they send me. I’m not gonna go do it. (Lebanese woman, 53—Participant 1)

**Table 3:** Participant-reported cultural barriers towards mammography.

Emerging Theme	Excerpt Number	Excerpt
Modesty	3.1	It’s like you’re saying—it’s more—that invasive part of your body where you don’t really want people to go—so if there’s no—if you see no problem and you think that—okay, I’m fine. Why go there? (Italian woman, 56—Participant 15)
	3.2	First of all, it’s humiliating. Okay, she’s a woman, all right, but it’s still a stranger. I don’t do that, not even with my bestest friends. I get embarrassed if we go to the beach together undressed. So you got to have this sensitivity. (Italian woman, 67—Participant 5)
	3.3	There’s always embarrassment. I mean, you can never sort of—even women to women, of course, it’s—you’re never at ease. (Italian woman, 57—Participant 2)
	3.4	I think it’s like just because that embarrassment, like having a pap smear, it’s the same like having breast—I think us, European women, we just think, “Oh, I will go next year,” and you don’t do it. (Croatian woman, 68—Participant 4)
	3.5	If it’s women doing—I prefer women—female doing it. I don’t like males doing it. I don’t mind seeing the male doctor, but when it’s got to do with just tests like that, I prefer female. (Italian woman, 70—Participant 11)
Language	3.6	Yeah the government send the letter to me. And they help me for Italian. You know...for my language (Italian woman, 75—Participant 7)
	3.7	My mother-in-law, she doesn’t speak English at all. Always she needs someone with her, her daughter or her son, anyone. She can’t speak, not even one word. And always she comments and say, “I don’t know this letter, what for.” I said “Mum, when I come to see you, I’ll read it for you.” (Lebanese woman, 53—Participant 1)
	3.8	Language. You know. I no understand everything. (Italian woman, 75—Participant 7)
	3.9	Sometimes—you know medical term is a bit harder—questions—so I can’t answer it, but if it is simple, I can answer it. (Fijian woman, 70—Participant 9)
No symptoms	3.10	There’s actually no history of breast cancer in my family and I haven’t had no indication that there’s anything going wrong and everything seems to be pretty normal. (Italian woman, 56—Participant 15)
Lack of awareness	3.11	I don’t think many of my friends—they’re all 50, but I don’t think they have been to those services yet….they’re not really—their awareness is low. (Pakistani woman, 53—Participant 16)
	3.12	Well, I know with the Maltese, they have the biggest morning tea and that’s—they collect money and thing for breast cancer. So that’s really big in our culture. When they’re having things like this, [they should use the opportunity to] make women more aware. (Maltese woman, 61—Participant 14)
Spouse permission	3.13	These things I have to talk to my husband first… you know… and then we decide all together… you know.. me and my husband. (Italian woman, 75—Participant 7)
Complementary medicine	3.14	They’re thinking—some of my friends do not believe in this [screening] and believe they can do natural medicine instead you know. (Italian woman, 75—Participant 10)

**Table 4:** Patient-reported personal barriers to breast screening.

Emerging Theme	Excerpt Number	Excerpt
Other conditions	4.1	I don’t wanna go. In my situation, I can’t do it. If they bruise, I’m not allowed to get the bruise. I get the bleeding and then I get sick. I don’t wanna do that. (Lebanese woman, 53—Participant 1)
Previous results	4.2	On that screening, they actually find that I did have something on the beginning of the breast. But they said, “Oh, there’s nothing to worry about ‘till maybe you’re 70. (Croatian woman, 68—Participant 4)
Self-examination	4.3	But I wouldn’t want to go, really, to screen. I already check every night, every day when I have my shower, every day. (Italian woman, 75—Participant 10)
	4.4	Like I said before, my fingers can feel it now. If there’s something there, I would run to you [doctors]. I would rather do it that way. (Italian woman, 75—Participant 10)
	4.5	I think I did try to examine myself to see if there’s anything there. If I find something, I would go but you think, “Oh, yeah, there is nothing there, so I’m all right.” Well, you’re not really expert but you just think, “Oh, it’s okay.”(Croatian woman, 68—Participant 4)
Age	4.6	I think up to a certain age, they do. Once you hit the 60 to 65, I’ve—through my experiences, women sort of say, “Look, I’m not gonna worry about it. What for? Everything’s been fine the last ten years. That’s it—I’m getting old. I am old. I’m not gonna bother. (Italian woman 57—Participant 2)
Fatalistic beliefs	4.7	I think sometimes it’s the fear, finding out. Yeah. They think it’s better if they don’t know. (Italian woman, 70—Participant 11)
	4.8	And some time—actually, they are scared to go there. They don’t want to know anything. Even if they—they don’t want to know. Maybe that’s why—the reason not going. (Pakistani woman, 53—Participant 16)
	4.9	But if you talk to some people, that’s another thing with my friends, they say, “Oh, if it’s gonna happen, it will happen anyway”. (Italian woman, 67—Participant 5)
Structural barriers	4.10	For me, it was the transport. My husband was working and it’s not that close to you to go. (Croatian woman, 68—Participant 4)
	4.11	The traffic and it’s so busy, so different—yeah, I didn’t like going there. (Italian woman, 67—Participant 5)
	4.12	Yeah, because I think you have to bother people. The people work and they’ve got a family. (Italian woman, 75—Participant 10)
	4.13	Otherwise, people are so busy today they’re not gonna think for themselves as much, are they? Do you know what I mean? Especially mothers, grandmothers—(Maltese woman, 72—Participant 12)
	4.14	Because they busy with their children, or they work, and they don’t give it time for life. (Uruguayan woman, 74—Participant 13)

**Table 5:** Patient-reported general facilitators to screening.

Emerging Theme	Excerpt Number	Excerpt
General practitioner	5.1	So if the doctor said, “Look, when is the last time you had your checks? I think it’s time for you to go” and give you a referral, then you go. You don’t have no choice but to go. (Croatian woman, 68—Participant 4)
	5.2	A lot of Middle Eastern women look at a GP as a little bit of a higher figure to anybody else. So, whether a friend would tell them, it will be, “Oh, yeah, I should.” But if a GP does make it an issue, I think they would. (Italian woman, 57—Participant 2)
	5.3	I think for me it’s easy, more easy, less embarrassing to go to the doctor. And I go to and he reminds me to do everything. (Uruguayan woman, 74—Participant 13)
BreastScreen letters	5.4	Ladies get the cancer... breast cancer mostly. So, when I receive a letter, I take my interest in to go and see a—go and do a screening for it. (Fijian woman, 70—Participant 9)
	5.5	Yeah, BreastScreen. That’s it, the pink envelope. They sent me a letter asking me to come and have the free breast mammogram done. So I went there and had it done. And then two years later, they sent me another letter reminding me that it’s due. So I went and I got it done again. (Pakistani woman, 53—Participant 16)
Friends and family	5.6	I got my brother who—specialist, not here but back home, and he always says, “Make sure you do all the right thing and check yourself and look after yourself. It’s important.” (Italian woman, 67—Participant 5)
	5.7	Yeah. Well, I’m starting to remind my daughter now ‘cause she’s coming of age now. Yes, I think so, mother-daughter thing. (Italian woman, 70—Participant 11)
	5.8	Every year, I might have breast cancer. My daughter, she says, “You need to go to the screening” for the breast cancer. (Uruguayan woman, 74—Participant 13)
	5.9	I grow up with the idea prevention is better than cure. So that’s how I was brought up by my grandparents, my parents, so better to know than not. (Italian woman, 67—Participant 5)
Group attendance	5.10	That would be a good idea too like if the ladies go in group, maybe they will support each other. They will think, “Okay, we have some support with us.” (Pakistani woman, 53—Participant 16)
Media	5.11	I came across one of my friend who had—recently who has—diagnosed with cancer. Since then, I’m more aware of it. In addition to that, on TV, Pink Ribbon and stuff—was a campaign also promote me to also raise my awareness of this. (Pakistani woman, 53—Participant 16)
	5.12	You get to hear a lot about famous people on television, like this one had it that one, and you go, “Oh, I should go.” But in family…women do not talk about it and you just put it behind you. (Croatian woman, 68—Participant 4)
Social media	5.13	I think the kids, yes. I think so ‘cause you have to think of them and kids maybe persuade the mother. (Maltese woman, 72—Participant 12)
	5.14	But if you put things on Facebook, everyone’s on it. (Maltese woman, 72—Participant 12)
	5.15	Social media is already playing some role, but I think they should play more than they’re doing at the moment. (Pakistani woman, 53—Participant 16)

## Data Availability

The data presented in this study are available on request from the corresponding author. The data are not publicly available due to privacy reasons.

## Appendix A. Interview Schedule

**Screening:**

- Have you had any breast cancer screening in the past?
- How many times have you attended?

**If have had screening:**

- When did you have that screening?
- Why did you decide to have that screening?
- Were there any difficulties you had to overcome to have the screening?
- Who advised you to have screening?

**If haven’t had screening:**

- Why did you decide not to have that screening?
- What healthcare encounters have you had in the past that may have influenced your screening decision?

**Understanding:**

- What is the purpose of screening?
- How regularly mammograms need to be repeated?

**Future screening:**

- How do you feel about getting breast cancer screening in the future?
- What information and support would encourage you to participate in future screening?
- How clear are the messages you see about breast cancer screening?
- What do you think could be done to increase cancer screening participation in general?
- Is there anything you would like to ask me?
